# Copper nanoparticles as a novel therapeutic approach for canine distemper virus: Clinical, hematological, and biochemical evidence from naturally infected dogs

**DOI:** 10.14202/vetworld.2025.2945-2954

**Published:** 2025-10-08

**Authors:** Ali Hussein Aldujaily, Douaa Barzan Salman, Kifah Fadhil Hassoon, Ghadeer Sabah Bustani

**Affiliations:** 1 Department of Veterinary Clinical Sciences, Faculty of Veterinary Medicine, University of Kufa, Kufa, Al-Najaf, Iraq; 2 Department of Veterinary Microbiology, Faculty of Veterinary Medicine, University of Kufa, Iraq; 3 Department of Anesthesia, Faculty of Medical Technologies, The Islamic University, Najaf, Iraq

**Keywords:** antiviral therapy, biochemistry, canine distemper virus, copper nanoparticles, hematology, nanomedicine, reverse transcription polymerase chain reaction

## Abstract

**Background and Aim:**

Canine distemper virus (CDV) remains a major cause of morbidity and mortality in dogs worldwide, particularly in unvaccinated populations. Current therapeutic options are largely supportive, with no widely available effective antiviral treatment. Nanotechnology-based therapies, such as copper nanoparticles (CuNPs), have recently shown promise against a range of viral pathogens. This study aimed to evaluate the clinical efficacy and safety of CuNPs in naturally infected dogs with CDV and to assess their effects on hematological and biochemical parameters.

**Materials and Methods:**

A total of 28 mixed-breed dogs (aged 2–8 months) were enrolled between January and February 2024. Clinical suspicion of CDV was confirmed by rapid immunochromatographic testing and reverse transcription polymerase chain reaction (RT-PCR) targeting the *N* gene. Fifteen CDV-positive dogs received oral CuNPs (0.5 mg/kg, twice daily for 5 days), while five untreated CDV-positive dogs served as controls; five healthy dogs were also included as negative controls. Hematological and biochemical parameters were monitored before and after treatment. CuNPs were characterized by ultraviolet–visible spectroscopy, scanning electron microscopy, and zeta potential analysis.

**Results:**

CDV was detected in 65% (15/23) of clinically suspected dogs by RT-PCR. Treated dogs demonstrated marked improvement in clinical signs, with complete recovery in all non-neurological cases and a 75% recovery rate in neurological cases. Hematological analyses revealed significant increases (p < 0.05) in red blood cell count, hemoglobin, packed cell volume, platelet count, white blood cell count, and lymphocyte count in CuNP-treated dogs compared with untreated controls. Serum biochemistry showed reductions in alanine aminotransferase, aspartate aminotransferase, alkaline phosphatase, blood urea nitrogen, and creatinine, alongside improved albumin levels after treatment. No adverse effects were observed during or after therapy.

**Conclusion:**

CuNPs demonstrated significant antiviral activity and clinical benefit in dogs naturally infected with CDV. The findings suggest that CuNPs may represent a promising adjunctive therapy for canine distemper. However, larger controlled trials are warranted to validate efficacy, optimize dosing, and ensure long-term safety.

## INTRODUCTION

Canine distemper (CD) is a severe systemic viral disease of dogs with global distribution, affecting multiple organ systems and often leading to fatal outcomes. The causative pathogen, canine distemper virus (CDV), belongs to the genus *Morbillivirus* in the family *Paramyxoviridae* and carries a single-stranded, negative-sense RNA genome [1].

Clinically, CDV infection primarily involves the respiratory, gastrointestinal, and central nervous systems. During the acute phase, affected dogs may present with cutaneous rash, oculo-nasal discharge, conjunctivitis, anorexia, secondary bacterial infections, and neurological manifestations [2].

Diagnosis can be challenging because the wide spectrum of clinical signs often mimics other diseases, making laboratory confirmation essential [3]. Commonly employed diagnostic tools include enzyme-linked immunosorbent assay for detecting virus-specific immunoglobulin (Ig)G and IgM antibodies [4]. However, polymerase chain reaction (PCR) is considered the gold standard due to its ability to directly detect viral genetic material in infected cells [5]. Current treatment strategies remain largely supportive and include broad-spectrum antibiotics, electrolyte replacement, respiratory support, and corticosteroids for central nervous system involvement. The use of antiviral agents is uncommon in veterinary medicine, and controlled data supporting their efficacy are scarce [3].

In recent years, nanotechnology has attracted attention as a novel approach to antiviral therapy for infections previously lacking effective treatment options. Among nanomaterials, copper nanoparticles (CuNPs) have shown promising antiviral activity against several viruses, including influenza A [6], herpes simplex virus [7], and hepatitis C virus [8].

CDV pathogenesis is mediated by two critical surface glycoproteins: Hemagglutinin (H), which facilitates viral attachment to host cell membranes, and the fusion protein (F), which promotes membrane fusion and viral entry [9]. The antiviral action of CuNPs is thought to involve multiple mechanisms: (1) Disruption of the viral envelope and subsequent degradation of viral RNA, (2) inhibition of key viral proteins, and (3) induction of reactive oxygen species (ROS), resulting in viral inactivation [10].

Despite vaccination efforts, CDV remains a major cause of morbidity and mortality in dogs worldwide, particularly in unvaccinated or partially immunized populations. Current treatment strategies are largely supportive, focusing on managing secondary bacterial infections and alleviating neurological or systemic complications. To date, there is no licensed antiviral therapy available for CDV, and controlled studies evaluating antiviral agents in veterinary medicine are scarce.

Recent advances in nanotechnology have highlighted CuNPs as promising candidates due to their demonstrated broad-spectrum antiviral activity against influenza, herpes simplex, and hepatitis viruses. However, most evidence supporting CuNP efficacy is derived from *in vitro* studies or experimental models of non-canine viruses. To the best of our knowledge, no systematic clinical evaluation of CuNPs in naturally infected dogs with CDV has been conducted. This creates a critical knowledge gap regarding their potential therapeutic benefit, safety profile, and ability to modulate clinical, hematological, and biochemical parameters in affected dogs. Addressing this gap is particularly important in regions where distemper remains endemic and treatment options are severely limited.

The present study was designed to investigate the therapeutic potential of CuNPs in dogs naturally infected with CDV. Specifically, the study aimed to (1) confirm CDV infection using rapid immunochromatographic testing and reverse transcription PCR (RT-PCR), (2) evaluate the clinical efficacy of CuNPs in improving survival and recovery rates, (3) assess their impact on hematological and biochemical parameters, and (4) explore their potential as a safe adjunctive antiviral therapy in veterinary practice. By providing the first clinical evidence of CuNP application against CDV, this study seeks to advance the development of nanomedicine-based antiviral strategies for companion animals.

## MATERIALS AND METHODS

### Ethical approval and Informed consent

This study was approved by the Kufa University Animal Experiments Local Ethics Committee (decision number 2024/62). Written informed consent was obtained from all dog owners before participation. All procedures were conducted under veterinary supervision in accordance with ethical guidelines.

### Study period and location

The study was conducted during January and February 2024. The study population consisted of 28 mixed-breed canine subjects (aged 2–8 months; male and female) clinically suspected of CDV infection, recruited from the Teaching Veterinary Hospital in Najaf and collaborating private veterinary clinics.

### Animal selection

Two groups were established:

1. Treatment group (n = 15): CDV-positive dogs whose owners consented to CuNP therapy.
2. Control group (n = 8): Dogs confirmed positive for CDV but not treated with CuNPs (n = 5), along with healthy controls (n = 3–5, depending on test type).

### Clinical assessment

Each dog was assessed for age, sex, weight, breed, and clinical signs of CDV, including neurological, gastrointestinal, respiratory, and ocular manifestations. Diagnosis was based on clinical signs, rapid antigen test results, PCR, and complete blood count. Dogs were classified as CDV-infected (n = 23) or healthy (n = 5).

### Blood sampling

Blood samples were collected from the cephalic vein using Ethylenediamine tetraacetic acid and plain tubes (BD Vacutainer, USA). For CDV-positive dogs, samples were collected on days 0 and 10 post-treatment. In healthy controls, samples were collected only once. Hematological and biochemical analyses were performed immediately.

### Hematological analysis

Red blood cell count (RBC), hemoglobin (Hb), packed cell volume (PCV), mean corpuscular volume (MCV), mean corpuscular Hb concentration (MCHC), platelet (PLT) count, white blood cells (WBC), lymphocytes, monocytes, neutrophils, eosinophils, and basophils were counted using an automated hematology analyzer (Genex, California, USA).

### Biochemical analysis

Serum biochemical parameters were determined using a Cecil CE 2041 series UV/Vis spectrophotometer (Cecil Instruments Ltd., Cambridge, England), in conjunction with commercially available diagnostic reagent kits supplied by Randox Laboratories Ltd. (Crumlin, County Antrim, United Kingdom; www.randox.com). All assays were performed according to the manufacturer’s instructions. The following were measured: Alanine aminotransferase (ALT), aspartate aminotransferase (AST), alkaline phosphatase (ALP), total bilirubin, total protein, albumin, blood urea nitrogen (BUN), and creatinine.

### Immunochromatographic assay

CDV antigens were detected in conjunctival swab samples using a commercial lateral flow rapid test kit (Rohi Biotech, China), following the manufacturer’s instructions.

### RT-PCR

#### Viral RNA extraction

Viral RNA was extracted from 20 blood samples using the AccuZol total RNA extraction kit (Bioneer, Korea). The protocol included chloroform separation, isopropanol precipitation, and RNA purity assessment with a NanoDrop 1000 spectrophotometer (Thermo Scientific, USA). A 260/280 ratio of ~1.8 was considered acceptable.

#### Primer design

Primers targeting the nucleoprotein (N) gene of Morbillivirus (GenBank accession no. EF375619.1) were designed using the Primer-BLAST tool provided by the National Center for Biotechnology Information (NCBI) primer design tool. The expected amplicon size was 591 bp.

- Forward: 5′-CAA GAG GAC TCG GGA CCA AC-3′
- Reverse: 5′-TCT CCG ACC ACA CGT CTT TG-3′

#### RT-PCR conditions

One-step RT-PCR was performed using the OptiScript RT system RT/PCR PreMix (iNtRON Biotechnology, Korea). Cycling conditions were as follows:

- Reverse transcription: 37°C, 30 min
- Initial denaturation: 95°C, 5 min
- 36 cycles of: denaturation (95°C, 30 s), annealing (58°C, 30 s), extension (72°C, 30 s)
- Final extension: 72°C, 5 min

PCR products were resolved on a 1% agarose gel (Thermo Fisher Scientific, USA), stained with ethidium bromide (Sigma-Aldrich, USA), and visualized under UV light using a Bio-Rad gel imaging system.

### Characterization of CuNPs

#### Source and preparation

CuNPs were obtained from the Faculty of Science, University of Kufa. For treatment, a solution was prepared by mixing CuNPs with polyvinylpyrrolidone (Sigma-Aldrich, USA).

#### UV-visible (UV-Vis) spectroscopy

**Figure 1:**
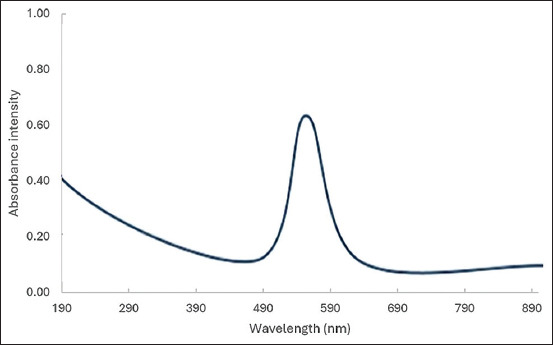
Ultraviolet-visible spectroscopy analysis of CuNPs. CuNPs = Copper nanoparticles.

Surface plasmon resonance (SPR) peaks were confirmed at 570 nm using a **UV-Vis spectrophotometer (Model: Cecil CE 2041, Cecil Instruments Ltd., Cambridge, United Kingdom), verifying successful nanoparticle formation. The UV-Visible spectrogram, as shown in Figure 1, was used to confirm CuNPs formation and to identify the surface plasmonic resonance peak at a wavelength of 570 nm.

#### Scanning electron microscopy (SEM)

**Figure 2:**
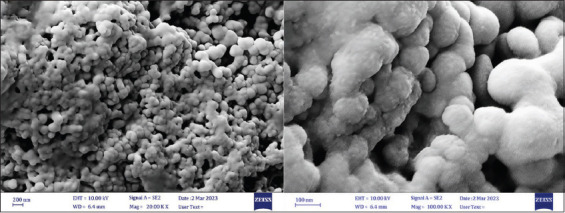
SEM analysis of the CuNPs. CuNPs = Copper nanoparticles, SEM = Scanning electron microscopy.

The morphology and size distribution of CuNPs were determined by SEM, revealing spherical, evenly dispersed particles with a mean diameter of 15.75 nm, as depicted in Figure 2.

#### Zeta potential analysis

**Figure 3:**
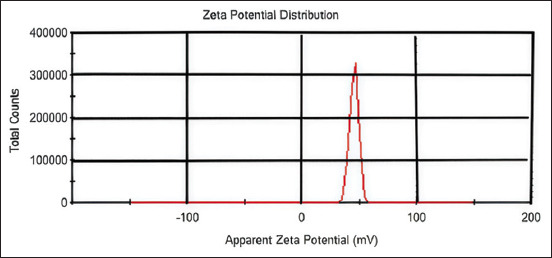
Zeta potential of the synthesized CuNPs at 42 mV. CuNPs = Copper nanoparticles.

The zeta potential of CuNPs was measured at 42 mV, indicating good colloidal stability, as shown in Figure 3.

#### CuNP treatment protocol

CuNPs were administered orally at a dose of 0.5 mg/kg twice daily for 5 consecutive days. Dogs with both neurological and non-neurological clinical signs were included in the treatment group.

### Statistical analysis

All statistical analyses were performed using IBM SPSS Statistics for Windows, Version 26.0 (IBM Corp., Armonk, NY, USA) and GraphPad Prism version 8.0.0 (GraphPad Software, LLC, San Diego, CA, USA). Data are expressed as mean ± standard error of the mean. One-way analysis of variance followed by the least significant difference test was used to compare differences among groups. A probability value (p-value) of <0.05 (p < 0.05) was considered statistically significant.

## RESULTS

### Clinical findings

Dogs naturally infected with CDV exhibited a diverse range of clinical signs, including fever, anorexia, prostration, ocular-nasal discharge, conjunctival congestion, diarrhea, vomiting, coughing, blindness, corneal opacity, tooth enamel hypoplasia, and hyperkeratosis. Neurological manifestations such as paddling, chorea, and muscle tremors were also observed (Table 1), which is consistent with a previous study by Yilmaz *et al*. [11].

**Table 1:** Clinical signs and treatment outcomes in canine distemper.

No.	Sex	Age	Clinical signs	After treatment
2	F	3 months	Gastrointestinal: Diarrhea and vomiting. Respiratory: Tracheobronchitis	Recovered
1	F	2 months	Ocular: Keratoconjunctivitis. Depression and weight loss	Recovered
3	M	6 months	Gastrointestinal: Diarrhea	Recovered
1	M	4 months	Respiratory	Recovered
2	M	3 months	Gastrointestinal disorders: Anorexia and depression	Recovered
2	M	4 months	Gastrointestinal: Diarrhea, Respiratory: Tracheobronchitis. Ocular: Keratoconjunctivitis	Recovered
1	M	2 months	Neurological gastrointestinal and respiratory	Dead
2	F	8 months	Neurological and gastrointestinal	Recovered
1	M	4 months	Neurological, gastrointestinal, and ocular	Recovered

Following treatment with CuNPs, most dogs showed marked improvement. Within 10 days, all but one dog recovered without sequelae; a single mortality occurred for unknown reasons.

Pantropic infection of CDV is known to target mononuclear phagocytes and lymphocytes, which act as transporters of the virus to epithelial tissues of the respiratory, digestive, dermal, and nervous systems, resulting in the observed pathological signs [3].

### Hematological findings

#### Erythrogram

**Figure 4:**
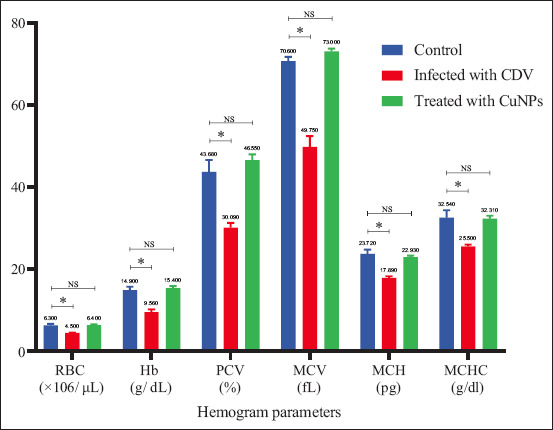
Hemogram parameters in control, CDV-infected, and CuNPs-treated groups. *Values are significantly different at the probability level (p ≤ 0.05). CuNPs = Copper nanoparticles, CDV = Canine distemper virus, RBC = Red blood cell count, Hb = Hemoglobin, PCV = Packed cell volume, MCV = Mean corpuscular volume, MCHC = Mean corpuscular hemoglobin concentration, MCH = Mean corpuscular hemoglobin, NS = No statistically significant difference.

**Figure 5:**
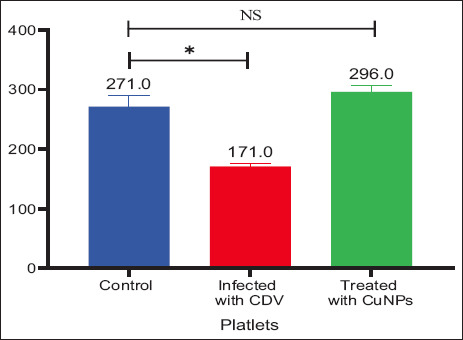
Platelets parameters in control, CDV-infected, and CuNPs-treated groups. *Values are significantly different at the probability level (p ≤ 0.05). CuNPs = Copper nanoparticles, CDV = Canine distemper virus, NS = No statistically significant difference.

Significant differences (p < 0.05) were observed in RBC count, Hb, PCV, MCV, mean corpuscular Hb, MCHC, and PLT levels between CDV-infected and healthy control dogs. These findings confirm that CDV can induce anemia, as previously reported by Saaed and Al-Obaidi [12]. Hematological parameters improved significantly in CuNP-treated groups compared with untreated infected dogs, though values remained lower than in healthy controls (Figures 4 and 5).

Decreases in PCV, Hb, and RBC values suggest erythroid hypoplasia [13] or interference with erythropoiesis through inflammatory mediators leading to reduced RBC lifespan [14]. Thrombocytopenia, frequently associated with viral infections, was also observed. Possible mechanisms include immune-mediated PLT destruction, direct viral effects on megakaryocytes, increased PLT consumption, and other depletion pathways [15, 16].

#### Leukogram

**Figure 6:**
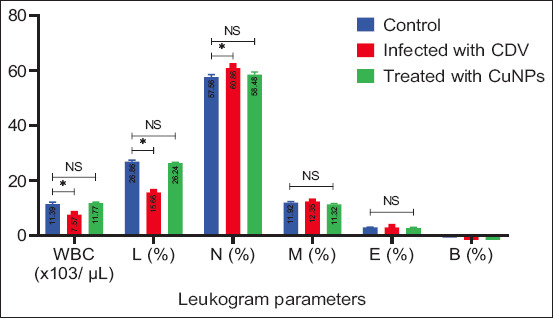
Leukogram parameters for control group, infected group with Canine distemper, and treatment group with CuNPs. CuNPs = Copper nanoparticles, WBC = White blood cells, NS = No statistically significant difference) CDV = Canine distemper virus. * = indicates a statistically significant difference (typically p < 0.05).

Leukogram analysis revealed significant reductions (p < 0.05) in total leukocyte and lymphocyte counts in infected dogs compared with healthy controls (Figure 6). Treatment with CuNPs resulted in a significant increase in WBC and lymphocyte counts.

Leukopenia and lymphopenia are hallmark findings in CDV infection and align with a previous study by Yama *et al*. [17]. These changes are typically due to viral replication between days 4 and 6 post-infection [18], along with CDV-induced apoptosis of lymphocytes [19]. Elevated granulocytes observed in infected dogs may reflect secondary bacterial infections and inflammatory responses [17]. The combination of leukopenia, lymphopenia, and neutrophilia highlights viral damage to lymphoid cells [20] and lymphoid depletion characterized by necrosis and apoptosis [21].

### Biochemical findings

**Figure 7:**
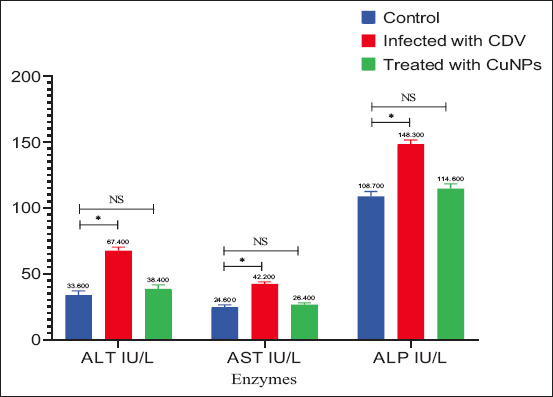
Liver enzymes for control group, infected group with canine distemper, and treatment group with CuNPs. CuNPs = Copper nanoparticles, NS = No statistically significant difference CDV = Canine distemper virus, ALT = Alanine aminotransferase, AST = Aspartate aminotransferase, ALP = Alkaline phosphatase. * = indicates a statistically significant difference (typically p < 0.05).

**Figure 8:**
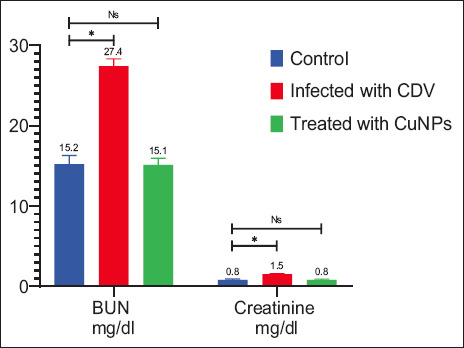
Creatinine and BUN for the control group, the infected group with Canine distemper, and the treatment group with CuNPs. CuNPs = Copper nanoparticles, NS = No statistically significant difference CDV = Canine distemper virus, BUN = Blood urea nitrogen. * = indicates a statistically significant difference (typically p < 0.05).

**Figure 9:**
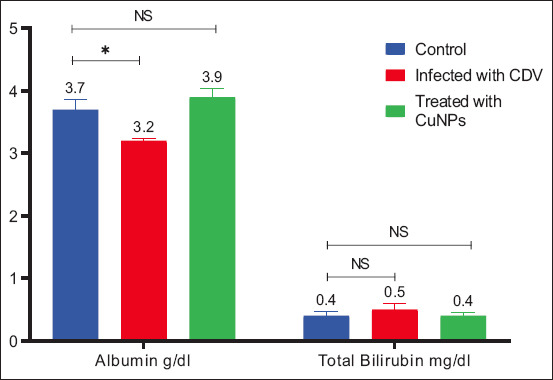
Albumin and bilirubin for control group, infected group with canine distemper, and the treatment group with CuNPs. CuNPs = Copper nanoparticles, NS = No statistically significant difference CDV = Canine distemper virus. * = indicates a statistically significant difference (typically p < 0.05).

Serum biochemical analysis revealed significantly increased liver enzyme concentrations (ALT, AST, and ALP), BUN, and creatinine levels in CDV-infected dogs, along with a significant decrease in albumin (Figures 7–9). These findings agree with a previous study by Saaed and Al-Obaidi [12]. Elevated ALP levels may result from gastrointestinal involvement in CDV [17], while high BUN and creatinine likely reflect dehydration [12]. The observed decrease in albumin further confirms the systemic impact of CDV infection.

### Virological findings

#### Antigen detection by immunochromatographic test

The rapid immunochromatographic assay detected CDV antigens in 52% (12/23) of clinically suspected cases. Clinical diagnosis alone was unreliable due to overlap with signs of other respiratory and gastrointestinal diseases. Conjunctival swabs, tracheal washings, and urine samples were useful for antigen detection, with conjunctival swabs being particularly sensitive due to continuous viral excretion in tear film [22–24].

#### Detection of CDV nucleocapsid gene by RT-PCR

**Figure 10:**
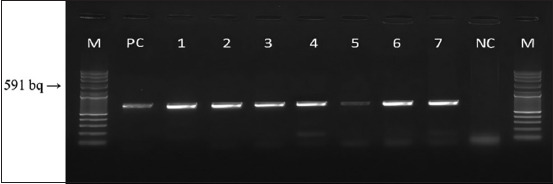
Agarose gel electrophoresis (stained with ethidium bromide) displaying the reverse transcription polymerase chain reaction products (591 bp) of the partially amplified *N gene of Morbillivirus* isolation. M represents the 100 bp DNA marker ladder; lanes 1–7 include positive control (PC), negative control (NC), and study samples 1–4, 6–7 (strong positive samples), with lane 5 showing a weak positive sample.

RT-PCR confirmed the presence of the CDV *N* gene (591 bp amplicon) in 65% (15/23) of samples (Figure 10). Among these, 11 dogs exhibited gastrointestinal, ocular, or respiratory signs without neurological involvement, while four dogs had neurological signs.

**Figure 11:**
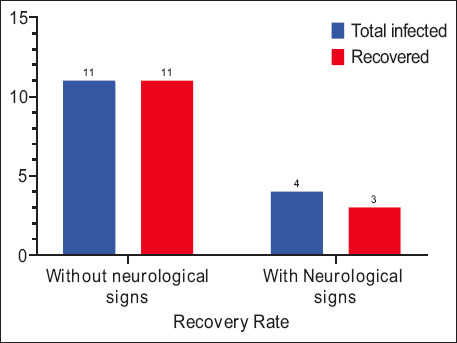
Recovery after treatment with CuNPs. CuNPs = Copper nanoparticles.

All dogs without neurological signs recovered completely (100%) after 10 days of CuNP treatment. Among the neurologically affected dogs, 3 recovered (75%), while one died of unknown causes. At the end of 3 weeks, all surviving treated dogs were clinically healthy and displayed no sequelae (Figure 11).

## DISCUSSION

### CDV in dogs

CDV is a highly contagious viral disease of dogs, particularly severe in unvaccinated young animals. Despite widespread vaccination campaigns, CDV continues to be reported in puppies that have received vaccines and in genetically predisposed wild and domestic species within the *Canidae* family [25].

### Diagnostic challenges

Clinical diagnosis of CDV is complicated by overlapping signs with other infectious diseases. Therefore, laboratory-based confirmation is essential. One-step reverse transcription polymerase chain reaction (RT-PCR) has been validated as a sensitive and reliable diagnostic tool for CDV detection [26, 27]. In the present study, *N* gene-based RT-PCR confirmed infection in 65% of tested samples, highlighting the active circulation of CDV in the region. However, as only clinically suspected dogs were sampled, this figure may not represent the true prevalence. Previous studies by Mansour and Hasso [28] and Al-Zaiyadi *et al*. [29] have reported higher CDV seropositivity rates, ranging from 26% to 46.6%.

### Interpretation of negative results

Dogs that tested negative by one-step RT-PCR may have harbored low viral loads, possibly corresponding to early infection, late infection, or the convalescent phase [30]. These results underline the importance of repeated testing and the use of multiple diagnostic methods for accurate disease confirmation.

### Vaccination and disease control

The detection of CDV in both vaccinated and unvaccinated dogs underscores the need for robust vaccination programs. Importation of fully vaccinated dogs into Iraq is strongly recommended to reduce the risk of introducing new CDV strains. Public awareness campaigns should also emphasize the importance of maintaining complete vaccination schedules in pet dogs to minimize the spread of disease.

### Potential role of CuNPs in antiviral therapy

Traditional antiviral drug development is often hindered by the diversity of viruses and their high mutation rates. CuNPs offer a promising alternative due to their broad-spectrum antiviral activity. CuNPs exert virucidal effects by disrupting viral capsids and envelopes [31, 32] and by generating ROS, which can inactivate viral particles [33].

### Immunomodulatory and broad-spectrum activity of CuNPs

Beyond direct antiviral action, CuNPs may enhance immune function by promoting the activity of T helper cells, B cells, and macrophages, leading to increased antibody production [34]. Their efficacy has been demonstrated against a wide range of viruses, including influenza [35], herpes simplex virus [36], Hepatitis B virus [37, 38], HIV-1, polio, and severe acute respiratory syndrome coronavirus-2 [33]. This broad-spectrum potential highlights CuNPs as a promising nanotechnology-based therapeutic platform for managing viral infections in both veterinary and human medicine.

## CONCLUSION

This study demonstrated the therapeutic potential of CuNPs in naturally infected dogs with CDV. Clinical outcomes showed rapid improvement in systemic and neurological signs, with complete recovery in all non-neurological cases and a 75% survival rate in dogs with neurological involvement. Hematological analysis revealed significant improvements in RBC count, Hb, PCV, PLT levels, and WBC in CuNP-treated dogs compared with untreated controls. Biochemical profiles also showed reduced ALT, AST, ALP, BUN, and creatinine levels, alongside improved albumin concentrations, suggesting restoration of liver and renal function. RT-PCR confirmed CDV infection in 65% of suspected cases, with CuNP treatment markedly enhancing recovery rates.

The findings suggest that CuNPs can serve as a novel adjunctive therapeutic option for canine distemper, a disease for which no effective antiviral treatment is currently available. Their dual antiviral and immunomodulatory properties may provide veterinarians with a low-cost, broad-spectrum tool for managing viral infections, particularly in resource-limited regions where distemper remains endemic. A key strength of this study is that it represents one of the first clinical investigations evaluating CuNPs in naturally infected dogs, integrating clinical, hematological, biochemical, and molecular diagnostics to provide comprehensive evidence of therapeutic benefit. However, the relatively small sample size, lack of long-term follow-up, and absence of a placebo-controlled design limit the generalizability of the findings.

Future large-scale, randomized controlled trials are essential to validate the efficacy and safety of CuNPs in CDV treatment. Research should also explore optimal dosing regimens, pharmacokinetics, and possible integration with existing supportive therapies. Moreover, extending such studies to other veterinary and zoonotic viral infections may broaden the application of CuNPs as a versatile antiviral platform.

CuNPs demonstrated significant clinical and laboratory benefits in dogs infected with CDV, offering promise as a novel nanomedicine-based therapeutic strategy. While preliminary, these findings open new avenues for integrating nanotechnology into veterinary antiviral therapy and ultimately contribute to improved animal health and welfare.

## AUTHORS’ CONTRIBUTIONS

AHA and DBS: Conceived and designed the study, drafted the manuscript, and revised it critically for important intellectual content. KFH: Contributed to data collection, performed part of the data analysis, and reviewed the manuscript. GSB: Conducted literature searches, participated in data interpretation, edited and revised the manuscript for intellectual content. All authors read and approved the final manuscript.
